# PET/CT Staging Followed by Intensity-Modulated Radiotherapy (IMRT) Improves Treatment Outcome of Locally Advanced Pharyngeal Carcinoma: a matched-pair comparison

**DOI:** 10.1186/1748-717X-2-22

**Published:** 2007-06-09

**Authors:** Sacha Rothschild, Gabriela Studer, Burkhardt Seifert, Pia Huguenin, Christoph Glanzmann, J Bernard Davis, Urs M Lütolf, Thomas F Hany, I Frank Ciernik

**Affiliations:** 1 Radiation Oncology, Zurich University Hospital, Rämistrasse 100, 8091, Zurich, Switzerland; 2 Medical Oncology, Kantonsspital Aarau, Tellstrasse, 5001, Aarau, Switzerland; 3 Institute for Social- and Preventive Medicine, University of Zurich, Hirschengraben 84, 8001 Zurich, Switzerland; 4 Radiation Oncology, Kantonsspital Graubünden, Loëstrasse 107, 7000 Chur, Switzerland; 5 Nuclear Medicine, Zurich University Hospital, Rämistrasse 100, 8091, Zurich, Switzerland; 6 Center for Clinical Research, Zurich University Hospital, Rämistrasse 100, 8091, Zurich, Switzerland; 7 Oncology Institute of Southern Switzerland, Ospedale San Giovanni e Valli, 6500 Bellinzona, Switzerland

## Abstract

**Background:**

Impact of non-pharmacological innovations on cancer cure rates is difficult to assess. It remains unclear, whether outcome improves with 2- [18-F]-fluoro-2-deoxyglucose-positron emission tomography and integrated computer tomography (PET/CT) and intensity-modulated radiotherapy (IMRT) for curative treatment of advanced pharyngeal carcinoma.

**Patients and methods:**

Forty five patients with stage IVA oro- or hypopharyngeal carcinoma were staged with an integrated PET/CT and treated with definitive chemoradiation with IMRT from 2002 until 2005. To estimate the impact of PET/CT with IMRT on outcome, a case-control analysis on all patients with PET/CT and IMRT was done after matching with eighty six patients treated between 1991 and 2001 without PET/CT and 3D-conformal radiotherapy with respect to gender, age, stage, grade, and tumor location with a ratio of 1:2. Median follow-up was eighteen months (range, 6–49 months) for the PET/CT-IMRT group and twenty eight months (range, 1–168 months) for the controls.

**Results:**

PET/CT and treatment with IMRT improved cure rates compared to patients without PET/CT and IMRT. Overall survival of patients with PET/CT and IMRT was 97% and 91% at 1 and 2 years respectively, compared to 74% and 54% for patients without PET/CT or IMRT (*p *= 0.002). The event-free survival rate of PET/CT-IMRT group was 90% and 80% at 1 and 2 years respectively, compared to 72% and 56% in the control group (*p *= 0.005).

**Conclusion:**

PET/CT in combination with IMRT and chemotherapy for pharyngeal carcinoma improve oncological therapy of pharyngeal carcinomas. Long-term follow-up is needed to confirm these findings.

## Background

Head and neck squamous cell carcinoma originating from the upper aerodigestive tract accounts for approximately 5% of all malignant tumors worldwide [1]. Surgical treatment can have debilitating consequences and radiotherapy with altered fractionation and concurrent chemotherapy have demonstrated improved treatment outcome [2-4]. By consequence, there has also been a change in the pattern of failure for this disease and fatal outcome from distant metastases in loco-regionally cured patients and the emergence of second primary tumors have become more common [5,6].

Clinical evaluation of primary site with aerodigestive endoscopy and cervical lymph nodes as well as palpation and measurement of cervical nodes are the basics. However, the incidence of false positive cervical nodes on clinical examination is approximately 15% [7]. Computer tomography (CT) and magnetic resonance imaging (MRI) provide a staging sensitivity ranging from 38% to 93%, respectively, with a specificity no higher than 71% to 83% [8,9]. Furthermore CT and MRI may lead to an incorrect staging of the primary tumor in up to 50% to 60% of the cases [10]. Positron emission tomography (PET) is sensitive and specific and allows metabolic mapping of cancer. Modern PET machines allow whole-body imaging, and are therefore, ideally suited to oncological imaging. The most widely used tracer, ^18^F-fluorodeoxyglucose (^18^FDG), acts as a glucose analogue allowing imaging of glucose metabolism, a process that is known to be enhanced in many malignant tumours [11]. Combining PET and CT has the potential to improve lesion localization, increase specificity, reduce interpretative pitfalls and to allow fast, low-noise attenuation correction, significantly increasing throughput. FDG-PET alone can provide up to 90% neck staging sensitivity and specificity [12]. A study on 202 consecutive patients at our department showed that FDG-PET has a major impact on the management of patients for radiotherapy, influencing both the stage and the management in 27% of patients [13]. When registered with CT images, the diagnostic accuracy may be further improved. The complex anatomy of the head and neck region poses difficulties in the interpretation of PET images without anatomical registration. Wong *et al*. have shown that CT or MR-PET-FDG images provide more clinically relevant information than obtained from clinical evaluation and conventional CT or MR staging [14]. Regarding the technical aspects of radiotherapy treatment planning, these studies conclude that PET/CT has made an impact on target treatment volumes in head and neck cancer [15-18].

Randomized studies on IMRT in head and neck cancer are lacking and a series of cohort data have been reported, all indicating high local an loco-regional control rates [19-21]. Intensity-modulated radiation therapy with an integrated boost (IMRT-IB) is a method of highly conformal therapy that combines several intensity modulated beams and achieves to treat two or more dose levels simultaneously [22]. The resultant isodoses are highly conformal even producing concave dose distributions [22,23]. IMRT and IMRT-IB has the ability to deliver high doses of radiation to the tumor with very high precision while minimizing the dose received by the surrounding normal tissues [24,25]. IMRT offers the potential for improved tumor control through delivery of high doses to the target volume with sharp dose gradients. This results in good sparing of surrounding normal structures [26]. Due to this advantages head and neck cancers are ideal sites for IMRT because the tumors often occur in close proximity to multiple critical normal tissues such as the brainstem, optic chiasm, optic nerves, parotid glands and spinal cord. Radiation doses that these critical organs can receive without causing complications lie in the range of 30–60 Gy [27]; however, the dose needed to control gross tumor often exceeds 70 Gy. Furthermore organ motion is virtually absent in the head and neck region, so daily patient setups can be reproduced accurately with adequate immobilisation. In the treatment of oropharyngeal carcinoma with IMRT there are several studies, which suggest favourable results for loco-regional tumor control and improved quality of life [26,28-30].

An clinical outcome analysis of PET/CT in combination with IMRT has not been reported so far, although broad interest exists for structured integration in clinical routine [31,32]. Both IMRT and integrated PET/CT have become available for treatment and treatment planning at the same time in our institution to be applied in a broad setting of cancer disease. To test each of the technological innovations within a controlled setting by itself in a randomized or at least controlled study is difficult in advanced cancer care. Thus, some doubt of the benefit of widely embraced novel technologies persists, especially as its use generally is associated with increasing health costs. It therefore is of major importance to estimate the additional value for cancer patients of PET/CT and IMRT.

## Patients and methods

### Patients

Between January 2002 and August 2005, 45 patients with locally advanced (AJCC stage IV) histologically confirmed squamous cell cancer of the oropharynx and hypopharynx underwent FDG- PET/CT for staging examination and IMRT treatment at the Department of Radiation Oncology (University of Zurich, Switzerland). Pre-treatment evaluation included a complete history and physical examination, direct flexible fiberoptic endoscopic examination, and complete blood counts. Pretreatment staging was done with ultrasound of the neck in all patients (100%) and all patients underwent a PET/CT scanning with diagnostic accuracy. A CT of the head and neck was available for 36 patients (82%), no PET, and MR was available for 9 patients (21%). For extra-regional staging, a CT of the chest was done in 2 patients (5%), CT of the abdomen in 1 cases (2%), ultrasound of the abdomen and liver in 1 patients (2%) and a bone scan was done in 0 patients (0%). PET/CT imaging and PET/CT-based RT planning was performed as described previously [16]. Briefly, No diabetic patients were accepted for PET/CT based RT-planning and injection consisted uniformly independent of body weight of 370 kBq 18-fluor-deoxy-glucose after fasting. Other examinations such as CT of the chest and abdomen, MRI scans of the head-and-neck region or 99mTc-diphosphonate bone scans were obtained in some patients when necessary. The disease was staged according to the 2003 American Joint Committee on Cancer staging classifications (AJCC) of 1997.

### Case-control matching

The forty five patients diagnosed and treated with modern technology were matched with patients without PET/CT and IMRT with respect to gender, age, AJCC stage, tumor grade, and tumor location (oro-/hyopharynx) using a ratio of 1:2. Selection bias was kept to a minimum by stringent matching of controls in chronologically reverse order from January 2002 accepting each matching patient. The 86 control patients were treated between January 1991 and December 2001. Patients in the control group underwent complete history, physical examination, direct flexible fiberoptic endoscopic examination, complete blood counts and radiological diagnostic (excluding FDG-PET/CT) and were treated with 3D-conformal radiotherapy after a dedicated CT has been obtained for treatment planning. Pretreatment staging of the control patients was ultrasound of the neck in all patients (100%), a CT of the head and neck, available for 75 patients (87%), PET was available for 16 patients (17%), and MR was available for 12 patients (14%). For extra-regional staging, a CT of the chest was done in 10 patients (12%), CT of the abdomen in 2 cases (2%), ultrasound of the abdomen and liver in 54 patients (63%) and a bone scan was done in 39 patients (45%).

All patients were treated with definitive radiotherapy. One female patient with a carcinoma of hypopharynx was not matched due to her age (34) at the time of diagnosis. Forty four cases with PET/CT and IMRT were matched with eighty six controls without PET/CT and 3D-conformal radiotherapy.

### Radiation treatment

Patients in the IMRT-group were treated up to a mean total dose of 69.0 Gy (range, 66.0 to 75.2 Gy). Treatments were administered in 30 to 35 fractions with 2.0 to 2.3 Gy given once daily.

In the control group the mean total dose was 71.5 Gy (range, 57.6 to 74.4 Gy). Most patients were treated during seven weeks, with 1.2 Gy given twice daily as described elsewhere[3] Briefly, the patients were treated with 1.2 Gy given twice daily with an interfraction interval of six hours. Volume definition and dose calculation were CT-based. Patients were treated with a head immobilization device. The radiation dose was prescribed according to the International Commission on Radiation Units and Measurements (ICRU 50 report)[33]. Shielding of the spinal cord after administration of 36 to 40 Gy limited the total radiation dose to this region to a maximum of 45 Gy from all fields. Spinal accessory lymph nodes were irradiated with electrons to a total dose of 50 Gy. For all locations, the Planning Target Volume (PTV) included the Gross Tumor Volume (GTV) with a margin of at least 2.5 cm to form the PTV up to 50 Gy; thereafter, the margin was reduced to 1.5 cm. Smaller margins were recommended if the GTV was close to organs at risk. The PTV was reduced twice; the recommended dose to involved areas was 74.4 Gy (72 Gy for volumes larger than 10 × 10 cm, 79.2 Gy maximum for smaller boost volumes excluding the larynx), whereas electively irradiated areas received 62 or 50.4 to 54 Gy, depending on the location relative to involved areas. There were no planned treatment interruptions.

**Figure 1:**
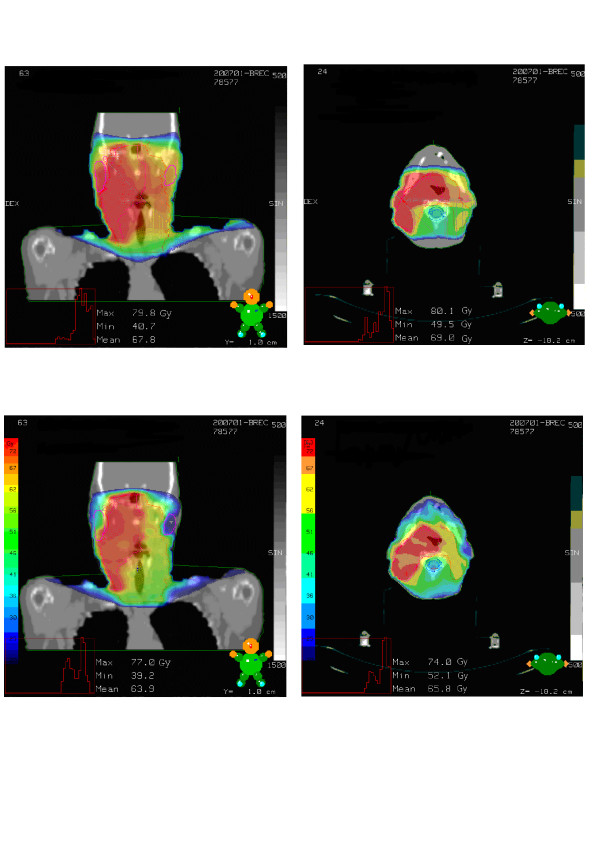
a. Example of a plan for 3D-conformal radiotherapy. b. Example of a plan for IMRT.

Figure 1 shows two patients with comparable disease treated with 3D-conformal radiotherapy (Fig. 1a) compared IMRT (Fig. 1b), respectively.

### Chemotherapy

All 45 patients in the PET/CT-IMRT group received concurrent chemotherapy with Cisplatin (CDDP) as a single agent. The scheme consisted of a planned five to seven weekly cycles of single agent CDDP (40 mg/m^2 ^body surface i.v.). thirteen patients (29%) had to discontinue chemotherapy before five cycles because of toxicity (two patients after one cycle, two patients after two cycles, one patient after three cycles, and eight patients after four cycles). Most of the patients had to discontinue the chemotherapy due to renal impairment or haematological toxicity.

In the control group sixty eight of eighty six patients (79%) received chemotherapy. Sixty three of them (93%) were treated with CDDP given as a single agent daily at a dose of 6 mg/m^2^. Other chemotherapy regimes comprised of Cetuximab (C225) in two patients (3%) composed of eight cycles of 450 mg, combination of taxol and paraplatin in a dosage of 130 mg and 250 mg respectively, applied in three cycles, in two patients (3%) and combination of 5-fluorouracil and cisplatin in a total dose of 9000 mg and 400 mg, respectively, in one patient (2%).

Adherence to chemotherapy was 71% in IMRT-group compared to 79% in the 3D-conformal group.

### Follow-up

All patients in both groups were evaluated at least once a week during radiotherapy. At the completion of radiation, the patients were then evaluated after six weeks by head and neck specialists, afterwards every three to six months. At each follow-up visit, a physical examination including a direct flexible fiberoptic laryngoscopy examination was performed. In most of the patients a CT or MRI scan of the head-and-neck region was performed. A FDG-PET/CT scan was also obtained in some patients in the PET/CT-group when clinically indicated.

### Statistical methods

Descriptive statistics (mean, median, proportions) were calculated to characterize the patient, disease, and treatment features. The differences in tumor characteristics between the PET/CT-IMRT group and the control group were examined by Fisher's exact test and Mann-Whitney U test using exact *p*-values. The 1- and 2-year event-free, local progression-free, regional progression-free, metastasis-free rates and overall survival probabilities were estimated using the Kaplan-Meier product-limit method. Differences were examined using the log-rank test. Durations were calculated from the date of diagnosis. The Cox proportional hazard model was used for multivariate analyses. *P*-values < 0.05 are considered statistically significant. All statistical analyses were performed using SPSS 13 (SPSS Inc.).

## Results

### Case-control matching accuracy

Table 1 shows the patient characteristics in both the PET/CT-IMRT and the control group. For the matched-pair analysis we excluded one female patient with age 34. The two groups were comparable. Age, gender and AJCC-stage were matched by design. Mann-Whitney test with exact *p*-values shows no significant difference in allocation of patients concerning T stage and grading. Separate inspection of T stages with Fisher's exact test shows a significant difference in T2 stages (27% vs. 11%, *p*-value 0.02). The influence of this significance on the outcome results has been ruled out with Cox-regression analysis, which doesn't change neither the hazard rate nor the *p*-value.

**Table 1:** Patient characteristics

	% Cases (n = 44)	% Controls (n = 86)	*p*-value
Age in yrs, median	56	56	Matched by design
Range	39–78	33–82	
Sex			
Male	80	80	Matched by design
Female	20	20	
Subsite			Matched by design
Mesopharynx	79	78	
Hypopharynx	21	22	
AJCC stage			
IV	100	100	Matched by design
T stage			0.20
T1	7	6	
T2	27	11	
T3	23	35	
T4	43	49	
Nodal status			0.70
N0	2	1	
N1	7	6	
N2a	7	6	
N2b	39	44	
N2c	43	41	
N3	2	2	
Grading			0.83
G2	55	63	
G2-3	7	8	
G3	27	28	
Missing	11	1	
Chemotherapy			<0.001
Yes	100	79	
No	0	21	

### Treatment

In the control-group there were significantly less patients receiving combined chemotherapy (*p *< 0.001). The statistical influence of this circumstance on outcome parameters was ruled out with Cox-regression analysis. Hazard rate and *p*-value don't change after correction for the Co-variant, as shown in table 2. When looking at the patients receiving chemotherapy there is no statistically significant difference between the IMRT-group were all patients were treated with cisplatin compared to the controls where 93% of the patients were treated with cisplatin and 7% with other regimens as mentioned above. The mean radiation dose in the two groups was statistically not different.

**Table 2:** Multivariate analyses for chemotherapy

**Control group**	**Source**	**Hazard rate (95% CI)**	***p*-value**
Event-free survival	Chemotherapy	2.2 (0.78–6.1)	0.14
	Group	0.28 (0.12–0.67)	0.004
Overall survival	Chemotherapy	0.76 (0.40–1.4)	0.40
	Group	0.15 (0.05–0.50)	0.002

### Outcome

**Figure 2:**
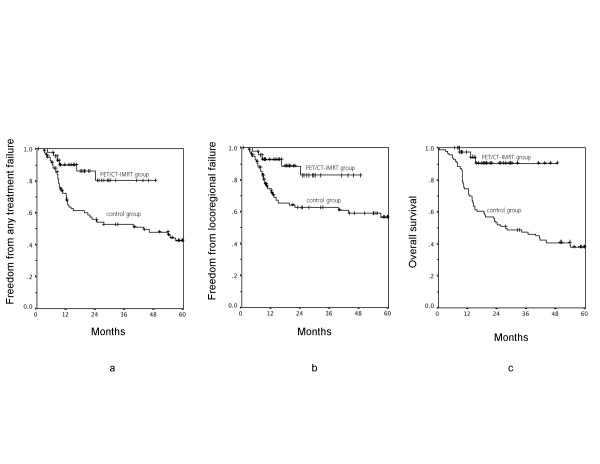
a. Time to any treatment failure (*p *= 0.005). b. Time to locoregional failure (*p *= 0.01). c. Overall survival (*p *= 0.002).

**Figure 3:**
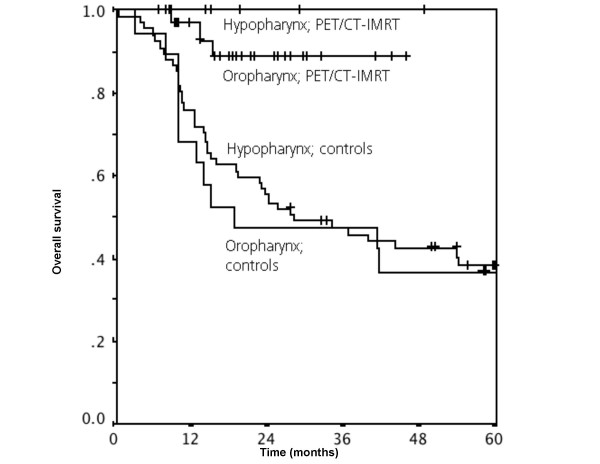
Overall survival for hypopharynx and oropharynx carcinoma (*p *= 0.02).

The median total follow-up time was eighteen months (range, 6–49 months) for the PET/CT-IMRT group and twenty eight months (range, 1–168 months) for the control group. All patients were followed for at least six months, as shown in figure 2 and 3.

The excluded patient in the PET/CT-IMRT group with age 34, is in complete remission twenty five months after the diagnosis.

### Time to treatment failure

Figure 2a presents Kaplan-Meier curves for time to any treatment failure. Time to any treatment failure was analysed by log-rank test. The failure-free rate at 1 year was 90% (SE 5%) in the IMRT-group and 72% (SE 5%) in the controls. At 2 years the failure-free rate was 80% (SE 8%) and 56% (SE 6%) respectively. The *p*-value for the Kaplan-Meier curve for event-free-survival is 0.005.

In the whole observation period there were five failures in the PET/CT-IMRT group. Four patients failed locoregionally, two patients only locally and two patients at the primary tumour site as well as at regional lymph nodes. Two patients presented with distant metastasis, from there one patient with concurrently locoregional and distant failure. Three patients died within the time of observation. The case of death was related to the malignancy. In the control group there were forty four failures. Twenty six patients had local and twenty one patients regional failure. Seventeen developed distant metastasis. Fifty six patients died, forty two related to the pharyngeal cancer.

### Local and regional tumor control

Patients in the novel technology group demonstrated a significantly higher rate of locoregional tumor control at 1 and 2 years. The locoregional failure-free rate at 1 year was 93% (SE 4%) and 73% (SE 5%) respectively. The rates at 2 year were 83%% (SE 8%) and 63% (SE 6%), respectively. The Kaplan-Meier curves are shown in Figure 2b with a *p*-value of 0.01.

Local control rates and regional control rates were also improved for PET/CT-IMRT-group, but this difference was not statistically significant.

### Overall survival

As shown in Figure 2c, overall survival at 1 and 2 years was 97% (SE 3%) and 91% (SE 5%) in the IMRT-group, compared with 74% (SE 5%) and 54% (SE 5%) in the controls (log-rank test, *p *= 0.002).

### Hypopharynx and oropharynx carcinoma

Looking at the data from hypo- and oropharynx carcinoma separately, there is a significant difference in event-free and overall survival for carcinoma of hypopharynx. The event-free survival at 1 and 2 years was 100% in the IMRT-group and 56% (SE 12%) and 45% (SE 12%), respectively in the controls, *p*-value 0.02. The overall survival at 1 and 2 years was 100% in the IMRT-group, compared with 68% (SE 11%) and 47% (SE 11%), respectively in the controls, *p*-value 0.04. These data are illustrated in figure 3.

In oropharyngeal carcinoma, a significant difference in the overall survival between 3D-conformal radiotherapy without PET/CT and PET/CT with IMRT was revealed. The overall survival at 1 and 2 years was 97% (SE 3%) and 89 % (SE 6%) in the IMRT-group, compared to 76% (SE 5%) and 55% (SE 8%), respectively. These data are also shown in figure 3 (*p *= 0.002). The event-free survival in the oropharynx carcinoma group missed significance concisely (*p *= 0.06).

## Discussion

The emergence of novel technologies frequently leads to changes of medical practice with respect to staging and work-up of cancer. Since the 1990s PET and since 2001 PET/CT have been used for staging and disease assessment[14,34,35], as well as for radiotherapy treatment planning. We and others have shown the feasibility of integrated-PET/CT-based radiotherapy planning and improved standardization of volume delineation compared with that of CT alone [16,18]. An increase in diagnostic accuracy resulting from the use of PET/CT has been suggested in several studies but the impact on therapy and potential improvement in cancer treatment has not been clearly demonstrated [36-38]. Investigations of diagnostic tools such as PET/CT for cancer staging or IMRT for cancer treatment are difficult to subject to prospective randomization. The reasons for this may be limited access to technology in many centres, the clear-cut theoretical advantages of innovative technology which make comparison to older technologies unattractive or ethically difficult, and the convenience provided by advanced technologies in daily practice. Additionally, technological improvements rarely enter the field sequentially, but as in case of PET/CT and IMRT, become available simultaneously. Thus the clinician is easy to convince, that the technological innovations such as PET/CT and IMRT will provide invaluable improvement in radiation oncology, despite lack of sound evidence exceeding phase II data for either PET/CT or IMRT. Indeed, randomized trial for PET/CT or IMRT for cancer patient treatment will remain difficult to conduct and may not be feasible, as the role of technology can not be easily disregarded in patient treatment planning and care. The only American randomized trial comparing 3D-conformal radiotherapy with IMRT had to be closed due to insufficient accrual (Dr. H. Lau, personal communication. Trial 20313 entitled (A randomized phase III study of conventional 3D-radiation vs. IMRT in SCCHN, NCT00363441). A french phase III trial (IMRT plus cisplatin versus conventional radiotherapy plus cisplatin in stage III-IV HNSCC) is run by the GORTEC and a British phase III trial, ICR-PARSPOT (parotid-sparing intensity-modulated radiation therapy compared with conventional radiation therapy) is addressing the question of reducing toxicity with IMRT. However, current toxicity data from single arm studies are likely to suffice to postulate a new standard with IMRT [17].

In the present paper, PET/CT in combination with IMRT strongly enhanced oncological treatment outcome of stage IVa pharyngeal carcinoma. We performed a case control study of patients in a limited time period over 15 years in order to evaluate outcome after RT. To our knowledge, the current study is the first in which the combination of PET/CT and IMRT and their impact on oncological treatment outcome is analysed. The results show an increase in event-free and overall survival. The data are striking and the differences in results from patients treated prior to 2002 and after with PET/CT and IMRT exceeded our expectancies.

There are several reasons for the positive impact of PET/CT and IMRT in the present series. Medicine has changed over the last decades and comparison of "modern" RT with historical cohorts is associated with bias, which can be hardly controlled for, such as staging and supportive care. First, the increased sensitivity and specificity of PET in the nodal staging of head and neck cancer has been well documented, and the anatomic information from the combined PET/CT can enhance the accuracy of staging [36-38]. More adequate staging may merely result in stage migration, because less advanced stages are preferentially treated and advanced metastatic stages might be detected with PET/CT and thus treated as palliative disease. PET/CT has been shown to improve staging quality resulting in stage migration lung cancer [34]. In the case of head and neck cancer, several recent studies on PET/CT leave undoubted the impact on treatment target volume delineation. In a study of 21 patients with nasopharyngeal or oropharyngeal primary tumors, Nishioka *et al*. [15] reported that PET/CT detected 39 positive nodes in contrast to only 28 nodes detected by clinical examination and CT or MRI, respectively. In four patients, the nodal status was increased, which resulted in modified target delineation and potentially avoiding geographic misses. Koshy *et al*. [39] analysed 36 patients with head and neck cancer and demonstrated an alteration in radiotherapy volume and dose in 14% and 11%, respectively. Clearly, the reported improved treatment outcome is not surprising, because correct nodal assessment *per se *will render treatment planning more accurate.

The second reason, why the results in the present study show improved outcome after PET/CT and IMRT may be due to IMRT itself. A recent phase II study demonstrated encouraging local control rates in patients with oropharyngeal carcinoma with 2-year estimates of local progression-free, regional progression-free, distant metastases-free, and overall survival of 98%, 88%, 84% and 98%, respectively [30]. Chao *et al*. reported on results after IMRT for Stage III and IV oropharyngeal tumors with a median follow-up of 33 month. The 4-year estimate of locoregional control was 87% and disease-free survival was 81%. These data suggest a clear benefit for IMRT [40]. Thus, reports, including the present series, with excellent oncological results can change clinical practice, despite lack of randomized data. Indeed, randomized trials investigating IMRT versus 3D-conformal radiotherapy are questionable in the presence of such data and not likely to be appreciated by the oncologists and patients. Level III evidence outcome data of IMRT thus still significantly add to the notion of reduced incidence and severity of treatment-related toxicity such as xerostomia or skin desquamation.

As to any retrospective cohort analysis, limitations to the present study exist. Treatment bias for IMRT can not be ruled out. It is possible, that patients with a very high local tumor burden requiring rapid initiation of radiotherapy were not offered IMRT due to longer planning time and they might have been treated with 3D-conformal radiation only. This is reflected in part by the seemingly higher proportion of T3 stage disease in the control group of patients treated without IMRT. The primary tumor volume is one of the major risk factors for treatment failure for 3D- conformal and IMRT [41]. Furthermore technical differences besides dose conformity come with IMRT. The IMRT technique used in all patients uses an integrated simultaneous boost (IB) technique, which may be have different biological intensity compared to conventional or accelerated treatment as used in the controls. Therefore, IMRT patients did likely benefit from biologically more aggressive treatment. However, this observation is interesting, because with IMRT, technological modifications immediately translate to biological modifications, seemingly beneficial.

Another limitation of this study is the simultaneous analysis for both PET/CT and IMRT. An attempt to define the impact on outcome for either technology alone was not possible due to the routine use of PET/CT during the last 4 years. Furthermore, the reasons of outcome "before" and "after" "new technology" radiotherapy analysed in respect of the two major improvements allow to be more precise in what over time allow patients to undergo better treatment. We can not be conclusive whether IMRT or PET/CT contribute most to the improved treatment outcome. The only evidence of the importance of IMRT relies in similar outcome data reported by others with IMRT alone. Furthermore we only analysed locally advanced stages of oro- and hypopharynx carcinomas. In how far early AJCC stage II and stage III disease may be better treated with PET/CT and IMRT remains to be investigated. In fact, currently our data with PET/CT compare well with other reports, without PET/CT planning. Therefore, it might well be, that the data in the present series are mainly due to IMRT and to a lesser extent to PET/CT [21].

Another issue favouring better results in the patients with modern treatment is the consequent use of cisplatin-based chemotherapy in recent years. A minor, however notable imbalance of the use of chemotherapy was noticed, although differences favouring modern treatment with PET/CT and IMRT persisted significant after correction for chemotherapy. Consequent use of chemotherapy emphasizes the value of modern oncological treatment strategies, as combined modality has been accepted to be superior to single modality radiotherapy for over a decade.

Furthermore, long-term follow-up is needed to confirm these findings, as metastatic disease progression may reduce the impact of loco-regional disease control. The outcome of other entities such as laryngeal carcinoma as well as oral cavity carcinoma remains to be defined with PET/CT and IMRT.

## Conclusion

Despite some limitations of the present case-control analysis, the present series strongly suggest, that proper implementation and integration of novel technologies in curative definitive radiotherapy of oro- and hypopharyngeal carcinoma improves oncological outcome with organ preserving oncological treatment. Novel technologies seem to have major potential for improving cancer treatment outcome. Cost-efficacy analyses are warranted to additionally clarify their broad application.
